# Thermo-Transient Receptor Potential Channels: Therapeutic Potential in Gastric Cancer

**DOI:** 10.3390/ijms232315289

**Published:** 2022-12-04

**Authors:** Gang-Fan Zong, Rui Deng, Su-Yun Yu, Ai-Yun Wang, Zhong-Hong Wei, Yang Zhao, Yin Lu

**Affiliations:** 1 Jiangsu Key Laboratory for Pharmacology and Safety Evaluation of Chinese Materia Medica, School of Pharmacy, Nanjing University of Chinese Medicine, Nanjing 210023, China; 2 School of Medicine & Holistic Integrative Medicine, Nanjing University of Chinese Medicine, No.138 Xianlin Avenue, Nanjing 210023, China; 3 Jiangsu Collaborative Innovation Center of Traditional Chinese Medicine Prevention and Treatment of Tumor, Nanjing University of Chinese Medicine, Nanjing 210023, China

**Keywords:** TRP, temperature, gastric cancer, ion channel, therapeutic target

## Abstract

Over the last decade, researchers have found abnormal expression of transient receptor potential (TRP) channels. In particular, members of the thermally sensitive subclass (thermo-TRPs) are involved in many disease processes. Moreover, they have a vital role in the occurrence and development of gastric cancer (GC). Accordingly, thermo-TRPs constitute a major pharmacological target, and the elucidation of the mechanisms underlying their response to physiological stimuli or drugs is key for notable advances in GC treatment. Therefore, this paper summarizes the existing literature about thermo-TRP protein expression changes that are linked to the incidence and progression of GC. The review also discusses the implication of such association to pathology and cell physiology and identifies potential thermo-TRP protein targets for diagnosis and treatment of GC.

## 1. Introduction

As of 2020, gastric cancer (GC) was ranked fourth among the world-leading causes of cancer-related deaths [1]. Almost two-thirds of patients with GC die from this disease. Despite efforts to improve diagnostic technology and patient management, the prognosis of GC patients has not improved significantly. The only available therapeutic intervention that provides a better prognosis for patients with GC is surgical resection [2]. One characteristic of GC that affects early diagnosis and renders therapeutic interventions ineffective is its asymptomatic phenotype. As a solution to these problems, scientists are inventing new treatments for GC patients and shifting their focus to personalized medicine.

Over the years, several treatment options have been tested and found to be successful to some extent. Some of these targeted therapeutic options include the antagonists of vascular endothelial growth factor (VEGF) therapy and antagonists of human epidermal receptor 2 (HER2) therapy [3]. Although these two therapeutic options have laid a foundation for more targeted and personalized medicine, challenges exist regarding the effectiveness of these treatments and how they influence the quality of life of the patient. As a result, scientists have recently diverted their focus toward the thermo-TRPs which are new potential therapeutic targets [4,5,6]. At present, thermo-transient receptor potential channels (thermo-TRPs) are related to many digestive system tumors. Excessive transient receptor potential cation channel subfamily V member 1 (TRPV1) expression has been linked with the development of colon and pancreatic cancers [7,8]. Moreover, immunohistochemical research has illustrated the expression of TRPV1 in colon adenocarcinoma [9]. Further studies have concluded that the concentration of a TRPV1 agonist known as extracellular polyamines in gastrointestinal tissues intensifies during cancer and inflammation. This implies that in colon cancer, TRPV1 is probably activated by polyamines and may contribute to cancer pain. Meanwhile, the phenomenon observed in zebrafish instigated the identification of unusual expressions and the migratory and proliferative roles of both transient receptor potential cation channel subfamily M (TRPM)7 and TRPM8 channels in pancreatic cancer [10]. In these gastrointestinal tumors, the high expression of thermo-TRPs in GC cells is worthy of our attention. For example, TRPM2 is expressed at the mRNA level in GC patients and is inversely associated with overall survival. Functional TRPM2 is highly expressed in the GC cell lines AGS and MKN-45, and its shRNA-mediated knockdown can inhibit proliferation and increase apoptosis. In addition, TRPM2 knockdown also sensitized AGS and MKN-45 cells to paclitaxel and doxorubicin treatment, leading to further reduction of cell viability [11,12]. Thermo-TRPs can affect cell differentiation, proliferation, migration and apoptosis by regulating the production of calcium ions (Ca^+^) and isoforms, thus affecting the carcinogenesis and development of GC [4,13,14,15]. Therefore, thermo-TRPs have an immense potential to become important biomarkers and effective therapeutic targets when personalized therapies are established for GC.

Targeting thermo-TRPs has the potential to inhibit the onset and development of GC. This makes thermo-TRPs potential targets for therapeutic intervention and prognosis prediction among GC patients. We therefore reviewed the existing literature on the association of thermo-TRPs and GC, and examined the mechanism involved in thermo-TRPs-mediated oncogenesis, as well as the approach for targeting thermo-TRPs in GC. The findings will offer crucial insight for clinical treatment as well as future research.

## 2. Thermo-TRPs

The first time that TRP channels were identified was in the insect, *Drosophila*. Mutations occurring in the TRP and TRPL genes generated selective depolarization caused by the influx of Ca^2+^ and sodium ion (Na^+^) [16]. As such, *Drosophila* mutants affect the light sensation and only light-induced TRPs are exhibited rather than a sustained light response [17,18]. In humans, the TRP channels extend throughout the respiratory system, immune system, peripheral nervous system, gastrointestinal system, central nervous system, cardiovascular system, and skin [19,20].

**Figure 1:**
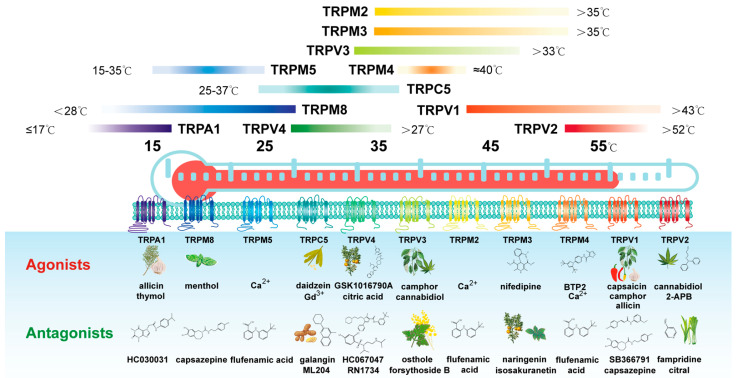
The activation of thermo-TRPs by specified temperature and particular compounds. Thermo-TRPs are expressed in diverse cell lines and are activated by ambient changes. TRPA1 is activated by noxious cold (≤17 °C). TRPM8, TRPM5, TRPC5, TRPV4, TRPM3, and TRPM2 are activated by innocuous warm and cool stimuli (ranges from 15–35 °C). Whereas TRPM4, TRPV1, and TRPV2 are activated by heat in the noxious range, with respective thresholds of 40 °C, 42 °C, and 52 °C. Moreover, all thermo-TRPs can also be activated by various agonists present in the environment. Thermo-TRPs, thermo-sensitive transient receptor potential channels.

Physiologically, TRP channels work as sensors for a wide range of cellular and environmental signals, as they can be gated by ligands, temperature, or mechanical stimuli (see Figure 1). A functional subset of TRP channels, including TRPV members TRPV1, TRPV2, TRPV3, and TRPV4 [21,22,23,24]; transient receptor potential cation channel subfamily A (TRPA) member TRPA1 [25]; TRPM members TRPM2, TRPM3, TRPM4, TRPM5, TRPM8; and transient receptor potential cation channel subfamily C (TRPC) member TRPC5, is distinguished by their sensitivity to temperature, named thermo-TRPs. The activation thresholds of different thermo-TRPs vary [26], ranging from noxiously hot to dangerously cold [27]. For instance, TRPV1 is activated by moderate heat (>43 °C), while TRPM8 is activated at 25–28 °C and below, ranging from cool to noxious cold. They sense different temperatures from comfortable warm (30–42 °C) and cool (15–30 °C) to painful heat (>43 °C) and noxious cold (<15 °C) [28]. They help many species, including mammals to avoid noxious temperatures and seek optimal thermal conditions [29]. The main characteristics of thermo-TRPs are summarized in Table 1, which mainly introduces thermo-TRP activation temperature, physiological functions, and correlation analysis between thermo-TRP expression and overall survival of GC patients. The above date in Table 1 are downloaded from the Kaplan-Meier plotter (http://kmplot.com/analysis, assessed on 15 September 2021).

## 3. Structures and Functions

**Figure 2:**
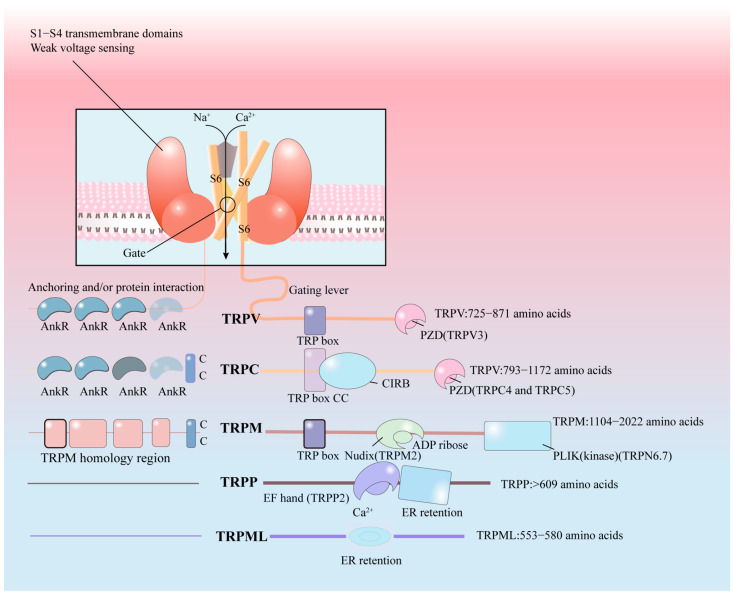
The TRP channels are composed of six transmembrane-spanning domains (S1–S6) that have a pore-forming loop connecting S5 and S6 as well as N and C termini situated intracellularly. In the S6 region, there are some structural differences between thermo-TRPs.

As per the current knowledge, the TRP channels are composed of six transmembrane-spanning domains (S1–S6) that have a pore-forming loop connecting S5 and S6 as well as N and C termini situated intracellularly. The selected cation penetrates through the pore ring on the outer surface of the cell, which is fixed by S5 and S6. Besides the similarities, there are some structural differences between them. (1) the C-terminal linked to S6 in TRPC contains a conserved 25-amino acid TRP domain; (2) in the N-terminal, the TRPC has ankyrin repeats of cytoplasmic domain; (3) TRPC, TRPM, and some TRPV family members have proline-rich regions between the C-terminal and S6 [16,30]. All TRP channels are non-selective except the monovalent selective channels TRPM4 and TRPM5, and the Ca^2+^ selective channels TRPV5 and TRPV6. The S1–S4 domains may bend relative to the S5-S6 domains in response to stimuli [16]. Different thermo-TRPs are unique in the S6 region (see Figure 2). Figure 2 highlights the diversity of cytoplasmic domains in thermo-TRPs. Various chemical and physical stimuli such as temperature changes, exogenous ligands, endogenous ligands such as Ca^2+^ and diacylglycerol, and stretch are known to influence these channels. Additionally, the depletion of intracellular Ca^2+^ has also been found to activate some of these channels [18].

**Figure 3:**
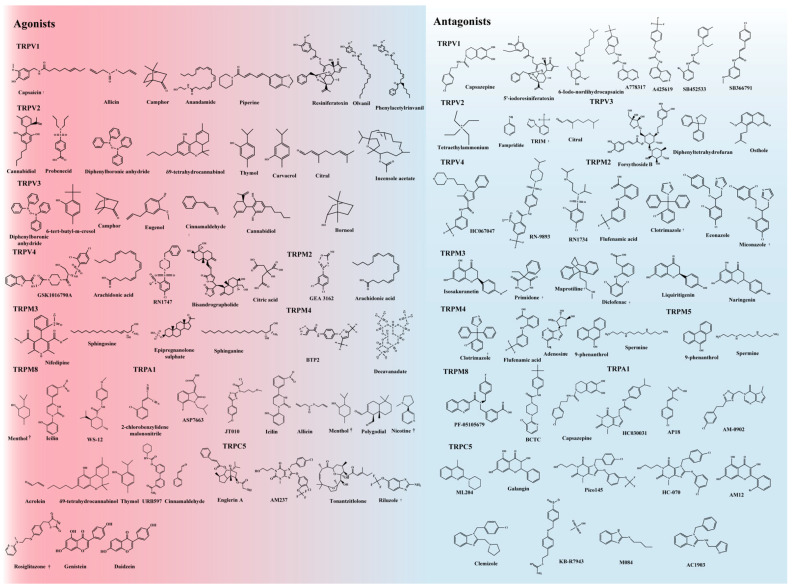
Representative agonist and antagonist structures of thermo-TRPs are approved by the FDA. In Figure 3, the agonists of thermo-TRPs are on the left in the red background, and the antagonists of thermo-TRPs are on the right in the blue background.

Thermo-TRPs are conducive to courses of taste, hyperalgesia, cenesthesis, pain, and mechanosensation. They are also involved in the adjustment of mucosal homeostasis, blood flow, and gastrointestinal motility. Due to their vital role in human pathology and physiology, thermo-TRPs have attractive potential as drug targets for cardiac remodeling, pain, anxiety, epilepsy, and cancer. Over the last few decades, several highly selective small-molecule agonists and antagonists of thermo-TRPs have been recognized as having potential pharmacological properties. The structures of representative agonists and antagonists of thermo-TRPs are depicted in Figure 3.

## 4. Thermo-TRPs Are Involved in Tumor Onset and Progression

### 4.1. Gastric Cancer

Many factors have been known to cause cancer with some factors being recognized as contributors to carcinogenesis while others are yet to be discovered. As per the current knowledge, *Helicobacter pylori* (*H. pylori*) is recognized as the most important risk factor associated with the development of GC [31]. *H. pylori* induce chronic inflammation which contributes to epigenetic modification of tumor suppressor genes thus allowing for uncontrolled cell proliferation, apoptosis evasion, and cell invasion [32]. It has been documented that TRPM2 ion channels can regulate macrophage polarization and gastritis during *Helicobacter pylori* infection [33].

Since thermo-TRPs are involved in numerous physiological processes that are vital for survival and cell growth, they have been dubbed a ‘Jack of all trades’, and this makes them potential candidates for cancer treatment. Specifically, the characteristics of thermo-TRPs that make them more suitable for cancer therapy can be illustrated by three points. First, some cancers have excessively expressed thermo-TRPs. Second, the activation of thermo-TRPs triggers Ca^2+^ signaling cascades associated with various features of cancer such as invasiveness, cell migration, apoptosis resistance, and proliferation. Third, the majority of thermo-TRPs are situated on the cell surface and this makes them easier targets for cancer therapies. Collectively, thermo-TRPs most likely affect cell differentiation, proliferation, migration, and apoptosis through the regulation of Ca^2+^ and the production of isoforms, therefore influencing the onset and development of tumors.

### 4.2. Cell Death and Proliferation

The induction of tumor cell death and inhibition of cell proliferation is an important strategy for cancer treatment. Proliferation is the initial stage of tumor development. The aberrant proliferation of tumor cells in situ ulteriorly promotes tumor metastasis and invasion progress and ultimately threatens the lives of patients. Therefore, the exploitation of tumor cell proliferation inhibitors plays an important role in reducing the harm caused by malignant tumors. If the proliferation balance of the cell is broken, the development of the cell from a normal differentiated state to a tumorigenic state involves changes in multiple key signaling proteins, thereby further developing into cells with a more aggressive phenotype. For the development of GC, this process involves a variety of thermo-TRPs expression and function changes [15].

#### 4.2.1. TRPV

More than one member of the TRPV family is linked to various cancer types including non-small cell lung carcinoma and breast cancer [34,35]. However, currently only TRPV2, TRPV4 and TRPV5 are identified as having a vital role in the development of GC. The expression of TRPV2 has been reported to be negatively correlated with the survival of GC patients [36,37]. Chemical inhibition of TRPV2 significantly augments cisplatin-induced death of GC cells, whereas overexpression of TRPV2 in GC cells weakens the killing effect of cisplatin. This uncovers the potential of TRPV2 as a therapeutic target for GC progression, and blockade of TRPV2 in combination with chemotherapeutic agents may boost the treatment efficacy for GC patients [37]. TRPV5 and TRPV6 comprise a distinct subfamily of homomeric and heteromeric channels found in transporting epithelia of the kidney and intestine [38]. They show strong inwardly rectifying currents and are the most Ca^2+^-selective TRP channels, and this sustained high Ca^2+^ concentration can kill cells through apoptosis and necrosis [15]. MKN45 cells were treated with GSK1016790A, a specific agonist of TRPV4, and then treated with RN1734, a specific antagonist of TRPV4, which could abolish the outwardly rectifying current, indicating the existence of specific TRPV4 current in MKN45 cells [39]. Other studies have indicated that TRPV4 function in GC cells is linked to G protein-coupled receptors (GPCRs) activation [40,41]. For instance, TRPV4 leads to a significant Ca^2+^ influx through vasoactive intestinal polypeptide receptor 1 (VPAC1) and calcium-sensing receptor (CaSR), both of which are GPCRs. Genetic silencing of TRPV4 expression or antagonist blocking effectively abolished VPAC1 and CaSR-related proliferation of GC cells [42]. Remarkably, the method involved in these phenomena differs based on the GPCR receptor’s function. TRPV4/CaSR promotes the survival of GC via AKT/beta-catenin/Ca^2+^ pathway whereas TRPV4/VPAC1 promotes GC cell survival through the ERK1/2/Ca^2+^/JNK/beta-catenin pathway. Although the findings from these studies provide compelling evidence concerning the crucial function of TRPV4 in the occurrence of GC, additional research is required to find out whether TRPV4 alone is biologically involved in GC, and whether targeting the function of TRP, alone or in combination with GPCR inhibitors, offers a viable solution for therapeutic intervention for GC. In addition, an epidemiological study has found that the agonist of TRPV1-capsaicin, is highly correlated to the presence of GC in the Mexican population [43].

#### 4.2.2. TRPM

Among thermo-TRPMs, TRPM2, TRPM3, TRPM5, and TRPM7 are known to contribute to the physiopathology of GC [44,45,46]. Maeda et al. indicated that excessive expression of TRPM5 is linked to the low survival rate of people with GC [47]. Regrettably, how TRPM5 affects the development of GC was not discussed in the research. However, it still shows the role of TRPM as a potential target for tumor progression. It was not until 2018 that it was first recognized that TRPM2 has an active role in GC. The researchers directed two shRNAs against TRPM2 to lower the level of its function and expression in the MKN45 and AGS GC cell lines. Their findings indicated that TRPM2 knockdown in GC cells decreases cell proliferation and enhances apoptosis. Moreover, they concluded that the absence of TRPM2 impairs cell autophagy, ultimately causing the death of cancer cells [11]. TRPM3 (permeable to Ca^2+^, Zn^2+^, and Mn^2+^) was the most recently identified ion channel of the TRPM family. TRPM3 activated by Ca^2+^ activates several downstream autophagy-related proteins (CAMKK2, AMPK, ULK1, LC3A, and LC3B), which promotes tumor growth [48]. In addition, miR-204, expressed from intron 6 of the human TRPM3 gene, targets Bcl-2 expression and enhances the responsiveness of GC [49]. TRPM7 is a rare Mg^2+^ channel, which is highly expressed in AGS and other GC cells [50]. Kim et al. inhibited GC cell growth significantly after inhibiting TRPM7 with ginsenoside Rg3 [51].

#### 4.2.3. TRPA

In GC, the available evidence leaves no doubt about the involvement of TRPA1. There is evidence that TRPA1 activation upregulates anti-apoptotic signals and improves the ROS tolerance of tumor cells. The inhibition of TRPA1 alleviated the growth of xenograft tumors and enhanced their chemical sensitivity [52]. However, its downstream represents a probable therapeutic target for diseases of the digestive system. Importantly, the potential therapeutic pathways involving TRPA1 downstream include bisphosphate-dependence, Phosphatidylinositol-4, and on account its calcium. In consequence, we can surmise that other Gq- or Gs-coupled or GPCR upstream agonists may also have failed to consider the TRPA1 component in their physiopathology. The neuropeptides calcitonin gene-related peptide (CGRP) and substance P appear to be implicated in the process of intestinal inflammation [53]. Oral cinnamaldehyde, an agonist of TRPA1, has the ability to reduce intestinal permeability in porcine epithelial cells [54]. Nevertheless, just a single clinical trial was done on volunteers who were healthy and considered capsaicin as an agonist of TRPV1 and cinnamaldehyde as an agonist of TRPA1. In addition, only data on capsaicin were published. From this, a TRPA1-based infection of inflammatory bowel disease may have a healing effect, although supportive clinical studies are not yet available. Meanwhile, further exploration is demanded of the relation between TRPA1 and GC, as well as the underlying mechanisms.

#### 4.2.4. TRPC

Existing literature has illustrated the expression of TRPC members in various types of cancer cells. For example, the expression of TRPC1, TRPC2, TRPC3 as well as TRPC4 have been found to affect the differentiation of the cells of non-small cell lung cancer. Though the thermo-TRPC member TRPC5 has been reported to be involved in glioma carcinogenesis [55], there is no evidence that TRPC5 is related to GC. However, a non-temperature sensitive member of TRPCs (TRPC6) is reported to be implicated in the progression of tumors. Cai et al. [56] found that TRPC6 expression was extraordinarily upregulated in the epithelial cells of GC in humans in comparison with the normal gastric epithelial cells. In addition, after SKF96365 treatment, an agent recognized for suppressing TRPC protection channels cell cycle was arrested in the G2/M phase, resulting in the suppression of cell growth. This may suggest that the site of activation of thermo-TRPs has a greater effect on tumor development.

### 4.3. Cell Migration, Invasion, and Metastasis

The metastasis of tumors is a crucial feature of cancer that denotes the fatal stage of tumor development, which contributes to 90 percent of cancer-related mortalities and is a great threat to patients’ lives. Unfortunately, very little is known regarding the molecular mechanism involved in the progression of metastasis. Before the cancer cells metastasize successfully, they have to first be able to migrate and invade the surrounding cells and local vasculature and then disseminate to further sites [57]. Conceptually, any changes in the metastatic cancer cells that invade the surrounding tissues or infiltrate the lymph and blood circulation have an effect on cell migration and invasion. With growing evidence indicating that TRP has an integral function in these processes, it is of great significance to illuminate the mechanisms of TRP in cell migration and invasion.

#### 4.3.1. TRPV

Some thermo-TRPV channels have been demonstrated to be involved in GC cell migration and invasion. The extracellular CaSR is widely expressed in the whole body, as the main regulator of calcium homeostasis, and abnormal CaSR function affects the development of cancer [58]. A recent study confirmed that the coupling of TRPV4 and CaSR promotes GC progression. It demonstrated that TRPV4 and CaSR were co-localized in GC cell lines MKN45 and SGC-7901, and that TRPV4 can be activated by CaSR, leading to Ca^2+^-induced cell migration and invasion [39]. In the meantime, Zhang et al. found the overexpression of the AKT gene may induce drug resistance in GC [59]. Besides, the TRPV1 channel, in spite of its role in temperature sensation, was also implicated in cell fate regulation. The expression levels of TRPV1 have been shown to be increased in prostate and bladder cancers [60,61]. Despite the lack of direct evidence that TRPV1 promotes the development of GC cell metastasis, epidemiological studies confirmed that high-dose capsaicin intake is closely related to the development of GC [43,62,63]. On the whole, these all together indicate that targeting TRPV channels might serve as prospective therapeutic strategies for GC.

Current research has indicated that TRPV1 is regulated and permeable for protons [64]. The acidic tumor microenvironment induces lymphangiogenesis and lymphatic metastasis via TRPV1 activation in lymphatic endothelial cells. Moreover, the expression of IL-8, one of the lymphangiogenic factors, was upregulated under acidic conditions [65]. Important to note is that during GC cell migration, upregulation of IL-8 could induce an epithelial-mesenchymal transition to accelerate tumor progression [66]. The drop in the extracellular pH can inhibit TRPV4-mediated Ca^2+^ influx in the oesophageal epithelial cells [67]. TRPV4 is highly expressed in GC [24]. CaSR activation induces TRPV4-mediated Ca^2+^ entry and further activates Wnt/β-catenin and PI3K/AKT pathways to exacerbate GC [39]. From this, we can see the pH levels of the skin, stomach, or tumors are neutralized through the acid-based homeostatic characteristics of these organs, and the confusion of cellular pH may lead to many diseases including tumors. Overall, when TRPV is activated by the tumor acidic microenvironment, the acidic environment required for tumor growth is further formed and releases tumor-promoting cytokines, leading to positive feedback and accelerating tumor progression.

#### 4.3.2. TRPM

Cell extracellular matrix (ECM) comprises basement membrane, intercellular substance, and multiple proteins. During tumor metastasis, a lot of reactions happened in tumor cells and ECM. Several regulatory pathways are either altered or aberrantly expressed, giving tumor cells the ability to accomplish metastasis [68]. Matrix metalloproteinases (MMPs) are considered to be involved in numerous processes of ECM-related tumor metastasis stages. Including tumor angiogenesis, tumor cell invasion, tumor cell intravasation, and tumor cell extravasation. Ren et al. found that the mRNA expression of MMP-13 in cancer tissues is significantly higher than those in normal tissues, and the high expression of MMP-13 shows significant relevance to tumor cell invasion and tumor stage [69]. At the same time, Laitinen et al. regarded serum MMP-8 as a prognostic biomarker in GC [70]. Currently, TRPM7 is the most reported member of the TRPM subfamily with the ability to regulate ECM. Rybarczyk et al. revealed that TRPM7 deficiency decreased the secretion of heat-shock protein 90α (Hsp90α), urokinase plasminogen activator (uPA), and pro-MMP-2, indicating TRPM7 affects the progression of pancreatic ductal adenocarcinoma (PDAC) by regulating the Hsp90α/uPA/MMP-2 proteolytic axis [71]. Activation of TRPM3 results in the enhancement of extracellular signal-regulated protein kinase (ERK1/2). Zhang et al. revealed the activation of NRAS/MEK1/ERK1/2 signaling facilitates tumor metastasis [72], and the expression of MMP2 is further upregulated. Although there is no direct evidence to confirm the effect of thermo-TRPM members on ECM in GC cells, there is evidence that functional expression of TRPM2 in GC cells promotes GC metastasis by inducing epithelial-to-mesenchymal transition (EMT) processes [73]. Considering that the main functions of ECM are structural scaffolding and biochemical support for cells and tissues, disease occurs when the homeostasis of the ECM is continually altered or disrupted. The EMT process continues to remodel the morphology and function of tumor cells, resulting in the loss of epithelial markers of tumor cells, changes in intercellular junctions, and enhance the detachment of cancer cells from situ, which are necessary for metastasis onset, but also persistent disruption of the ECM homeostasis. Since the effect of TRPM2 on EMT has been reported, we suspect that TRPM may regulate GC metastasis by affecting ECM. Activation of TRPM3 results in the enhancement of extracellular signal-regulated protein kinase (ERK1/2).

TRPM is associated with various changes in microenvironments, including cellular pH, hypoxia, and cytokines [74,75]. TRPM2 could be repressed via extracellular acidification at a pH of 6.5 due to the competition between protons and cations such as Na^+^ or Ca^2+^. Additionally, the depletion of TRPM2 results in the suppression of HIF-1α signaling, which is highly correlated with the hypoxic microenvironment [76,77]. It has been found that HIF-1α is crucial for the attainment of GC cells migration ability [78]. Furthermore, HIF-1α was described as a transcription factor mediating cell metastasis. Additionally, the activity of TRPM8 is reduced by extracellular protons. It was speculated that TRPM8 might influence tumor progression by interacting with the tumor cell microenvironment. In addition, TRPM8 inhibition leads to a pro-inflammatory cytokine profile in macrophages [79]. Account of the accumulation of various cytokines in the tumor microenvironment frequently triggers TRP channel-dependent signaling cascades [80]. Accordingly, it is significant to explore the influence of TRPM on tumor metastasis from this perspective of microenvironments.

#### 4.3.3. TRPA

The increased production of reactive oxygen species in cells is highly associated with hypoxia and impacts tumor development [81]. TRPA1 is activated by reactive oxygen species (ROS), naturally, TRPA1 could be exploited for targeted ROS-related cancer therapies [82]. Plenty of evidence has shown the role of ROS in GC metastasis. There is research found that increased ROS generation impairs the migration of GC cells. Meanwhile, Sun et al. demonstrated the generation of intracellular ROS in cells regulated GC migration both in vitro and in vivo [83]. Therefore, TRPA has the potential to study ROS-related GC metastasis.

#### 4.3.4. TRPC

The Ca^2+^-permeable cation channels are the mammalian affiliates of the classical TRP channel subfamily that have a role in the increase of receptor-mediated intracellular Ca^2+^ [84]. Intriguingly, numerous Ca^2+^-dependent mechanisms of malignant migration have been parsed. The Ca^2+^ signal is integral in regulating immune cell function, and the immune system may either be critical to the destruction of cancer cells or promote pro-metastatic pathways [85]. C-C motif chemokine ligand 18 (CCL18) is an example of the intersection between immune cells and cancer cells. The migration of cells, as well as, their invasion was dose-dependently enhanced by CCL18 stimulation [86]. Moreover, the Ca^2+^ signal can also be key in how immune cells destroy cancer cells [87]. The expression of TRPC1/3 was upregulated in GC cells in response to the TGF-β1-induced EMT, and blockade of TRPC1/3 channels with nonspecific pharmacological inhibitors attenuated EMT progression by suppressing the Ras/Raf1/ERK1/2 signaling pathway [88]. However, the previous publication also confirmed that the expression of TRPC3 is not associated with GC [46]. Considering the different GC cell lines used in both experiments, the discrepancy in conclusions is reasonable. This also reminds us to lay emphasis on the integrity of the experimental design.

## 5. Discussion

GC presents a significant health challenge due to its high incidence, dismal prognosis, and limited therapeutic options. While there has been a decline in the age-adjusted mortality rate for these diseases over the last few years, further research is needed to examine the underlying mechanism of anti-gastric cancer effects. It has already been established that ion channels are implicated in various diseases and biological processes. Specifically, various Ca^2+^ channels have been associated with the behavior of cancer cells and the physiopathology features of cancer. Any variations in the expression or activation of Ca^2+^ transporting proteins result in changes in the Ca^2+^ homeostasis and might modify localized Ca^2+^ signals and Ca^2+^ microdomains, which in turn affect Ca^2+^ dependent signaling process associated with tumorigenesis. While the majority of the Ca^2+^ channels have not been linked to cancer cells and may be found in many normal tissues, some are excessively expressed in cancer cells.

Over the last two decades, many studies have illustrated that TRP channels are involved in diverse cancer types, making them earn the name oncochannels. Among these channels, the thermo-TRPs, which are sensitive to changing temperature, have been considered to be emerging targets of GC therapy. Therefore, we discussed the physiological and pathological effects of thermo-TRPs. In addition, various thermo-TRPs are expressed differently in the same types of cancer. As per the existing evidence, many channel types may contribute to and interact within a single tumorigenic process, or play a role at various phases in the sequenced events involved in the process of tumorigenesis. Nevertheless, these studies do not infer that any single channel is associated with the occurrence of specific types of cancer, and it is unclear whether such a specific association exists.

We summarized the role of thermo-TRPs in various stages from initiation to metastasis of GC. We recognized that thermo-TRPs are frequently mutated in GC. Therefore, drug development targeting thermo-TRPs has potential significance for the development of anti-gastric-cancer therapies. However, although several specific agonists and antagonists of thermo-TRPs have been developed, there is a lack of commercially available low-toxicity and efficient drugs hitherto. Therefore, it is urgent to conduct further research on thermo-TRPs and develop more suitable targeted drugs to serve the clinical treatment of GC. Furthermore, in addition to the currently known pathway of thermo-TRPs affecting the development of GC, the target thermo-TRPs function in other digestive system tumors and diseases should also be of concern. This could lead to new directions for developing drugs for GC therapy. In addition, it is absolutely imperative to evaluate the overall biological effects prior to potential clinical application to avert the effect of thermo-TRPs on other stromal cells.

**Figure 4:**
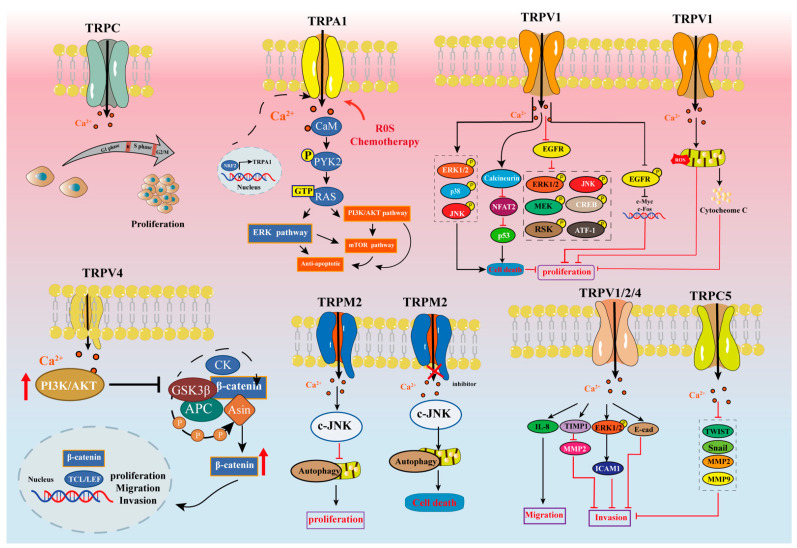
Mechanism of gastric cancer proliferation, invasion and metastasis mediated by representative thermo-TRPs.

Above all, the research on TRP channels is still in its infancy since it is a relatively new research field. See Figure 4 for the summary of the mechanism of thermo-TRPs in GC in the stages of proliferation, invasion, and metastasis reported so far. Accordingly, we investigated the role of thermo-TRPs in GC, hoping to pave the way for major clinical progress in therapeutic inventions for GC.

## Figures and Tables

**Table 1:** Different members of the Thermo-TRP and their main characteristics.

Channel Subunit	Activation Temperature	Physiological Functions	Association with Patient Outcomes
**TRPV subfamily**			
TRPV1	>43 °C	Thermo-sensation (heat); autonomic thermos regulation; inflammatory hyperalgesia; nociception and pain management	negatively associated with the overall survival of GC patients (*p* < 0.0001)
TRPV2	>52 °C	Thermo-sensation (noxious heat); nociception; mediated immune response	negatively associated with the overall survival of GC patients (*p* < 0.0001)
TRPV3	>33 °C	Thermo-sensation (moderate heat); nociception; skin integrity, hair growth and sebocyte function, mood regulation	negatively associated with the overall survival of GC patients (*p* = 0.015)
TRPV4	>27 °C	Thermo-sensation (moderate heat); mechano-sensation; osmo-sensation; nociception; endothelium vaso-motor control and shear stress sensor; modulation of cell migration; control adherens junctions in skin	negatively associated with the overall survival of GC patients (*p* < 0.0001)
**TRPM subfamily**			
TRPM2	>35 °C	Thermo-sensation (moderate heat); oxidative and nitrosative stress response; immunity cells infiltration; regulation of pancreas insulin release; apoptosis control	negatively associated with the overall survival of GC patients (*p* = 0.00054)
TRPM3	>35 °C	Regulation of pancreas insulin release and glucose homeostasis; steroid hormone (pregnanolon) sensor	negatively associated with the overall survival of GC patients (*p* < 0.0001)
TRPM4	~40 °C	Regulation of catecholamine release from chromafn cells; involved in mast cell activation and dendritic cell migration; regulation of Ca^2+^ entry	negatively associated with the overall survival of GC patients (*p* = 0.015)
TRPM5	15–35 °C	A key component of taste (sweet, bitter, umami) transduction; regulator of glucose-induced insulin release	negatively associated with the overall survival of GC patients (*p* < 0.0001)
TRPM8	<28 °C	Thermo-sensation (cold); autonomic thermos regulation (with TRPV1)	negatively associated with the overall survival of GC patients (*p* = 0.0015)
**TRPA subfamily**			
TRPA1	≤17 °C	Thermo-sensation (noxious cold); mechano-sensation; chemo-sensor; nociception; inflammatory pain	negatively associated with the overall survival of GC patients (*p* < 0.0001)
**TRPC subfamily**			
TRPC5	25–37 °C	Control of anxiety, fear and reward behaviors; promotion of brain development (together with TRPC1)	negatively associated with the overall survival of GC patients (*p* < 0.0001)

## Data Availability

Not applicable.
